# Advancing Antimony(III) Adsorption: Impact of Varied Manganese Oxide Modifications on Iron–Graphene Oxide–Chitosan Composites

**DOI:** 10.3390/molecules29174021

**Published:** 2024-08-25

**Authors:** Huinan Mo, Huimei Shan, Yuqiao Xu, Haimin Liao, Sanxi Peng

**Affiliations:** 1 College of Environmental Science and Engineering, Guilin University of Technology, Guilin 541004, China; mohuinan00612@163.com (H.M.); 15078696626@163.com (Y.X.); lhm208619@163.com (H.L.); 2 Collaborative Innovation Center of Water Pollution Control and Water Security in Karst Area, Guilin University of Technology, Guilin 541004, China; 3 College of Earth Science, Guilin University of Technology, Guilin 541004, China

**Keywords:** Sb(III) adsorption, manganese oxide, graphene oxide–chitosan composites

## Abstract

Antimony (Sb) is one of the most concerning toxic metals globally, making the study of methods for efficiently removing Sb(III) from water increasingly urgent. This study uses graphene oxide and chitosan as the matrix (GOCS), modifying them with FeCl_2_ and four MnO_x_ to form iron–manganese oxide (FM/GC) at a Fe/Mn molar ratio of 4:1. FM/GC quaternary composite microspheres are prepared, showing that FM/GC obtained from different MnO_x_ exhibits significant differences in the ability to remove Sb(III) from neutral solutions. The order of Sb(III) removal effectiveness is MnSO_4_ > KMnO_4_ > MnCl_2_ > MnO_2_. The composite microspheres obtained by modifying GOCS with FeCl_2_ and MnSO_4_ are selected for further batch experiments and characterization tests to analyze the factors and mechanisms influencing Sb(III) removal. The results show that the adsorption capacity of Sb(III) decreases with increasing pH and solid–liquid ratio, and gradually increases with the initial concentration and reaction time. The Langmuir model fitting indicates that the maximum adsorption capacity of Sb(III) is 178.89 mg/g. The adsorption mechanism involves the oxidation of the Mn-O group, which converts Sb(III) in water into Sb(V). This is followed by ligand exchange and complex formation with O-H in FeO(OH) groups, and further interactions with C-OH, C-O, O-H, and other functional groups in GOCS.

## 1. Introduction

Antimony (Sb), a common heavy metal, is widely used in mining, metallurgy, electronics, and fireproof materials [1]. The resulting wastewater causes serious water pollution if discharged untreated [2]. Therefore, developing efficient and economical antimony pollution control technology is a key focus in current environmental science research. Traditional heavy metal removal methods, such as chemical precipitation, ion exchange, and reverse osmosis, are widely used, but they have issues like high cost, serious secondary pollution, and unstable treatment efficiency [3,4,5]. In comparison, adsorption technology is an effective method to treat heavy metals in water due to its simple operation, low cost, and high efficiency [6]. Among various adsorption materials, composites based on graphene oxide (GO) and chitosan (CS) gain significant attention for their chemical stability and excellent adsorption properties. Graphene oxide, with its unique two-dimensional structure and high specific surface area, offers numerous adsorption sites for pollutants [7]. Chitosan, a natural biopolymer, also performs excellently in environmental treatment due to its multifunctional properties and good biocompatibility [8]. The iron-modified graphene oxide chitosan composite combines the high adsorption capacity of iron with the properties of graphene oxide, efficiently capturing heavy metal ions through a stable chemical structure and multifunctional active sites. Xiong et al. [9] graft the carboxylic metal–organic framework (MOFs) of iron oxide nanoparticles (nano-Fe_3_O_4_) and MIL-100 (Fe) onto chitosan (NMCS) and investigate its adsorption of Sb(III) in water. The results show that the maximum removal efficiency of Sb(III) at pH 11 is 96.8%, much higher than that of nano-Fe_3_O_4_ or MOFs.

As a common transition metal, manganese (Mn) compounds with different valence states possess unique chemical and physical properties. Shan et al. [10] find that introducing MnO_x_ significantly improves the adsorption capacity of Fe@GOCS composite for As(III), and the modification effect of some specific MnO_x_ is more significant. Additionally, while Mn as a common transition metal has been extensively studied in environmental science, research on the chemical modification of Fe-GO-CS composite materials using different MnO_x_ and their effects on the adsorption performance for Sb(III) remains relatively limited. Mn compounds in different oxidation states, such as divalent manganese salts (MnSO_4_, MnCl_2_), tetravalent manganese (MnO_2_), and heptavalent manganese (KMnO_4_), possess unique chemical and physical properties that improve adsorption performance through distinct mechanisms. Mn(II) is active at lower redox potentials and can enhance the removal efficiency of heavy metals through ion exchange and surface adsorption processes [11,12]. Mn(IV), due to its high oxidizing power, can form stable composite oxides on the material surface, not only enhancing the adsorption capacity for Sb(III) but also potentially converting Sb(III) into a more adsorbable or removable form through oxidation reactions [13]. Mn(VII), especially potassium permanganate, with its strong oxidizing nature, can not only improve the oxidation state of the material surface but also facilitate more effective pollutant removal through oxidation reactions, particularly for organic contaminants and recalcitrant substances [14]. As an element in the same family as As(III), Sb(III) is similar to As in form and chemical properties [15]. It can be inferred that Fe/Mn/GO/CS also has good adsorption and removal performance for Sb(III), but no reports exist on this research domestically or internationally. Research on optimizing its performance through further surface modification, especially using different manganese salts for chemical modification, is still relatively limited.

In this study, Fe@GOCS is used as the basic framework and chemically modified with different MnO_x_ (FM@GC) to investigate the adsorption properties and mechanism of Sb(III) on the modified material. The adsorption effect of FM@GC modified with different MnOx is compared through experiments, analyzing the influence of the type and dosage of MnOx on the adsorption efficiency of Sb(III). The potential mechanism of the adsorption process is explored through adsorption isotherm and kinetic studies. Additionally, the surface properties of the materials and their interaction with Sb(III) are analyzed using FTIR, XRD, and SEM.

## 2. Materials and Methods

### 2.1. Materials

All reagents including ferrous chloride tetrahydrate (FeCl_2_∙4H_2_O), manganese chloride tetrahydrate (MnCl_2_∙4H_2_O), potassium permanganate (KMnO_4_), manganese sulfate (MnSO_4_), hydrochloric acid (HCl), and sodium hydroxide (NaOH) of analytical grade were purchased from Xi Long Scientific Co., Ltd. (Shanghai, China). Antimony potassium tartrate trihydrate (C_8_H_4_K_2_O_12_Sb_2_∙3H_2_O), a source of Sb(III), was obtained from Macklin Biochemical Co., Ltd. (Shanghai, China). Graphene oxide was sourced from Jiangsu CF Graphene Technology Co., Ltd. (Suzhou, China), and chitosan was procured from Xi Long Chemical Co., Ltd. (Shantou, China). Throughout the research, deionized water (18.2 MΩ∙cm) was prepared using a Milli-Q water system (Millipore, Burlington, MA, USA). A stock solution of 1000 mg/L Sb(III) was prepared by dissolving C_8_H_4_K_2_O_12_Sb_2_∙3H_2_O in deionized water and further diluted to the concentrations required for batch experiments.

### 2.2. Preparation of FM @ GC Composite Materials

The FM@GC composite material loaded with FMBO was synthesized according to our previous studies with some modifications as follows [10,16]: 0.4 g of graphene oxide powder was added to 100 mL of 1.5% acetic acid solution. The mixture was then placed in a beaker and subjected to simultaneous ultrasonic treatment and stirring for 40 min to fully disperse the graphene oxide. Subsequently, 2.0 g of chitosan powder was added, and the mixture was ultrasonically heated and stirred until the chitosan completely dissolved. Afterward, FeCl_2_·4H_2_O, MnCl_2_·4H_2_O, KMnO_4_, MnSO_4_, and MnO_2_ were added to the mixture in a 4:1 ratio, and stirring continued until complete dissolution to obtain the Mn/Fe/GO/CS mixed solution. This mixture was then dropped into a 7% NaOH solution to form beads approximately 3 mm in diameter. After standing in the dark at room temperature for 24 h, the beads were filtered from the NaOH solution, washed until the wash liquid was nearly neutral, and then placed in 100 mL of 5% glutaraldehyde–formaldehyde mixed solution. The beads were reacted under water bath oscillation at room temperature (180 rpm) for 6 h for crosslinking, followed by repeated washing with deionized water until neutral. The beads were then dried in a forced air oven at 45 °C to constant weight, producing dark brown composite microspheres of FM@GC approximately 1 mm in diameter.

### 2.3. Batch Adsorption Experiment

For the batch adsorption experiment, 50 mg of FM@GC composite microspheres was placed in a 100 mL centrifuge tube, to which 50 mL of 10 mg/L Sb(III) solution was added. The pH of the solution was adjusted to 3.0 using NaOH and HCl solutions, and the mixture was incubated in a constant temperature water-bath oscillator for 2.0 days at 25 °C and 180 rpm. At the end of the reaction, 9 mL of the supernatant was taken using a 10 mL disposable syringe and filtered through a 0.45 μm filter. The concentration of Sb(III) was then measured using an inductively coupled plasma optical emission spectrometer (ICP-OES), and the adsorption equilibrium concentration *C_e_*, removal efficiency *R_e_*, and adsorbed amount *Q_e_* were calculated according to Equations (1) and (2), respectively.

(1) $$R_{e}=\frac{C_{0}-C_{e}}{C_{0}}\times 100\%$$

(2) $$Q_{e}=\frac{C_{0}-C_{e}}{m}\times V$$

where *R_e_* is the removal rate of the target pollutant at equilibrium (%), *C*_0_ is the initial concentration of Sb(III) (mg/L), *C_e_* is the concentration of Sb(III) at equilibrium (mg/L), *Q_e_* is the adsorption capacity (mg/g), *V* is the volume of the solution containing Sb(III) (L), and m is the mass of the adsorbent (g).

For the isotherm adsorption experiment, 50 mg of FM@GC composite microspheres were placed in a 100 mL centrifuge tube, to which 50 mL of Sb(III) solution with initial concentrations of 10, 50, 100, 150, 200, 250, 400, 500, and 700 mg/L was added. The pH was adjusted to 3, and the samples were incubated in a constant temperature water-bath oscillator at 25 °C for 48 h. Afterward, the solution was filtered through a 0.45 μm filter to measure the Sb(III) concentration. The data were fitted using the Langmuir and Freundlich isotherm models:

(3) $${Langmuir:Q}_{e}=\frac{Q_{m}K_{L}C_{e}}{1+K_{L}C_{e}}$$

(4) $$Freundlich:Q_{e}=K_{F}C_{e}^{1/n}$$

where *Q_e_* is the adsorption capacity at equilibrium (mg/g), *C_e_* is the concentration of Sb(III) at equilibrium (mg/L), *Q_m_* is the maximum adsorption capacity of the material for Sb(III) (mg/g), *K_L_* is the Langmuir equilibrium constant related to the strength of adsorption interactions, and *K_F_* and 1/*n* are the Freundlich equation constants for adsorption equilibrium and intensity, respectively.

For the adsorption kinetics experiment, 50 mg of FM@GC composite microspheres were placed in a 100 mL centrifuge tube, and 50 mL of 35 mg/L Sb(III) solution was added. The pH was adjusted to 4.0, and samples were taken at intervals of 10, 30, 60, 120, 180, 300, 420, 600, 780, 1020, 1260, 1560, 1860, 2280, 2700, 3240, 3780, 4320, and 4980 min. At regular intervals, solutions were transferred from separate tubes at the corresponding time points, filtered through 0.45 μm filters, and the concentration of Sb(III) was measured.

The total adsorption rate of the adsorbent can be controlled by one or more steps. To study the adsorption rate and behavior during the adsorption process, pseudo-first-order kinetics (see Equation (5)) and pseudo-second-order kinetics (see Equation (6)) were applied to simulate the adsorption kinetics of Sb(III) on the adsorbent. The pseudo-first-order kinetic model is based on the membrane diffusion theory and assumes that the adsorption process is controlled by physical adsorption [17]. The pseudo-second-order kinetic model assumes that the adsorption process involves the sharing or transfer of electron pairs between the adsorbent and the adsorbate, and is determined by chemisorption [17]. The Weber–Morris intraparticle diffusion model (see Equation (7)) was used to identify the rate-controlling step in the adsorption process. This model assumes that the rate-controlling step of adsorption is determined by intraparticle diffusion, rather than surface adsorption or hydrodynamic factors [17]. The equation is as follows:

(5) $$\log\left(Q_{e}-Q_{t}\right)=\log Q_{e}-\frac{k_{1}}{2.303}t$$

(6) $$\frac{t}{Q_{t}}=\frac{1}{k_{2}Q_{e}^{2}}+\frac{t}{Q_{e}}$$

(7) $$Q_{t}=K_{ip}t^{0.5}+C$$

where *Q_e_* (mg/g) and *Q_t_* (mg/g) are the adsorption amounts of Sb(III) at equilibrium and reaction time *t*, respectively. *K*_1_ and *K*_2_ are first-order and second-order rate constants respectively. *K_ip_* is the intragranular diffusion model constant, and *C* is the thickness of the surface boundary layer. If the fitted lines for *Q_t_* and *t*^0.5^ pass through the origin (*C* = 0), it indicates that intraparticle diffusion is the rate-controlling step. If the fitted line for *Q_t_* and *t*^0.5^ does not pass through the origin (*C* ≠ 0), it suggests that factors other than intraparticle diffusion are influencing the adsorption process.

### 2.4. Analytical Techniques

The concentration of Sb(III) in aqueous solution was determined by an inductively coupled plasma optical emission spectrometer (Optima 7000DV, Platinum Elmer Instruments, Inc. Waltham, MA, USA). Surface morphology and elemental analyses of Fe/Mn-GOCS were determined by JSM-7900F SEM-EDS (JEOL, Tokyo, Japan). The IS10 FTIR spectrometer (Thermo Fisher, Waltham, MA, USA) was used to determine the functional groups of Fe/Mn-GOCS. The crystal structure of Fe/Mn-GOCS was determined by X’Pert3 powdered multifunctional XRD (Panaco, London, United Kingdom, copper target, λ = 1.54056 Å). The scanning step, speed, and range were 0.02626°, 0.6565°/s, and 5°–90° (2*θ*), respectively.

## 3. Results and Discussion

### 3.1. Characterization

#### 3.1.1. XRD Analysis

**Figure 1:**
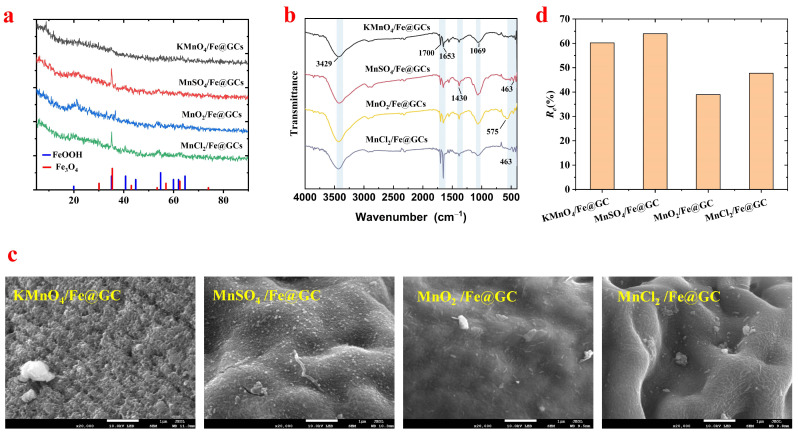
XRD (**a**), FTIR (**b**), and SEM (**c**) images of Fe@GC modified with different MnOx, and a comparison of adsorption capacities for Sb(III); comparison of removal effects for different MnO_x_-modified FM@GC on Sb (III) (**d**).

Figure 1a illustrates the XRD patterns of FM@GC composites modified with different MnO_x_. It can be observed that the FM@GC composites with the addition of KMnO_4_ exhibit amorphous characteristics. The FM@GC composites modified by MnSO_4_ and MnCl_2_ show a distinct sharp characteristic peak at 2*θ* = 35.11°, indicating the formation of a crystal structure, and smaller broad peaks at 2*θ* = 22.11°, 40.66°, and 53.92°, similar to the standardized FeOOH (PDF 26-0792) and Fe_3_O_4_ (PDF 26-0792) characteristic peaks, suggesting that the corresponding phases may be formed [14,18]. Additionally, the MnO_2_-modified FMGCs show a broad peak at 2*θ* = 21.17° and small sharp characteristic peaks at 33.23°, 36.65°, and 53.79°, similar to the characteristic peaks of FeOOH (PDF 26-0792), suggesting that these composites may contain these hydroxide forms [19]. However, the MnO_x_ is not clearly observed in all FM@GC, which could be attributed to two possible reasons: (1) Mn content in the Fe-Mn binary oxides is low relative to the Fe content due to the relatively low initial Fe/Mn reactant moles used in all the reaction systems. (2) The presence of Mn oxides is in amorphous form [18]. These XRD results not only reveal the crystal structure characteristics of the materials, but also provide a basis for further understanding the effects of different MnO_x_ modifications on the structure and properties of FM@GC composites.

#### 3.1.2. FTIR Analysis

In the Fourier transform infrared spectroscopy (FTIR) results shown in Figure 1b, FM@GC composite microspheres modified by different MnO_x_ (KMnO_4_, MnCl_2_, MnSO_4_, MnO_2_) exhibit their unique chemical structures. All samples exhibit a broad O-H hydroxyl group vibration peak at 3429 cm^−1^, corresponding to the -OH groups in GO and CS [20]. Additionally, the C=O vibration peaks at 1700 cm^−1^ and 1653 cm^−1^ suggest the presence of -COOH groups [8]. The key C-N vibration peak at 1430 cm^−1^ is significantly correlated with the type of MnO_x_ in each sample [8]. Fe/KMnO_4_@GC shows a weaker peak, which may reflect the effect of the strong oxidation of KMnO_4_ on the amino structure of CS. Conversely, Fe/MnO_2_@GC peaks at this wave number are significantly enhanced, suggesting a strong interaction between MnO_2_ and -NH_3_ of chitosan. In addition, the Mn-O bond vibration peaks at 463 cm^−1^ and 575 cm^−1^ in Fe/MnCl_2_@GC- and Fe/MnSO_4_-modified samples indicate the interaction between the chemical integration of MnO_x_ and the Fe@GC substrates [21]. It can be seen that the type and introduction of MnO_x_ have a decisive effect on the chemical properties and functional properties of FM@GC composites.

#### 3.1.3. SEM Analysis

SEM images of FM@GC with different MnO_x_ modifications are shown in Figure 1c. The surface of KMnO_4_/Fe-GC shows a rough and irregular surface with a pronounced bumpy and porous structure. This structure may result from partial degradation of the material surface due to the strong oxidation by KMnO_4_, enhancing the surface area and improving adsorption performance. This rough surface may increase the contact area with contaminants. Similar to the KMnO_4_ sample, the surface of Fe/MnSO_4_@GC is not smooth and shows many white particles, indicating that MnSO_4_ induces different crystal growth or deposition on the material surface. These particles may be reaction products of MnSO_4_ with CS and GO, providing more active sites and enhancing adsorption capacity. The surface of Fe/MnO_2_@GC is observed to have white needle-like structures, presenting a certain degree of roughness. Combined with XRD analysis, FeOOH is speculated to form on the surface of Fe/MnO_2_@GC, indicating a possible partial oxidation of Fe(II) in the presence of MnO_2_, resulting in the formation of FeOOH. The presence of FeOOH may further enhance the chemical stability and adsorption capacity of the material, especially for those contaminants removable by redox reactions. The modification of Fe/MnCl_2_@GC may promote smoother crystal growth on the surface, resulting in different adsorption properties, especially for larger molecules or where finer surface structures are required. SEM images of various manganese salt-modified Fe/Mn@GOCS composite microspheres reveal different surface morphologies directly related to their respective chemical treatments and the nature of the MnO_x_. KMnO_4_ and MnSO_4_, due to their higher oxidative properties, tend to form rougher and more porous structures on the composite surfaces, enhancing their adsorption capacity. In contrast, the smoother surfaces produced by MnO_2_ and MnCl_2_ may be favorable for adsorption in specific situations.

#### 3.1.4. Adsorption Capacity Analysis

To investigate the effects of different MnO_x_ on the adsorption of Fe/Mn@GC, the adsorption properties of Sb(III)) for various MnO_x_-modified composites are studied. The results are shown in Figure 1d. The removal efficiency of Sb(III) by Fe/MnO_2_@GC and Fe/MnCl_2_@GC is only 38.98% and 47.74%, while that by Fe/MnSO_4_@GC and Fe/KMnO_4_@GC is 63.96% and 60.20%, respectively. In conclusion, compared with Fe/Mn@GC modified by four different MnO_x_, the material modified by MnSO_4_ has more advantages in the adsorption of Sb(III) and the highest removal efficiency among the four modified materials. In summary, Fe/MnSO_4_@GC is selected for the follow-up experiment and is referred to as FM@GC for brevity.

### 3.2. Influencing Factors

#### 3.2.1. Influence of pH Value

**Figure 2:**
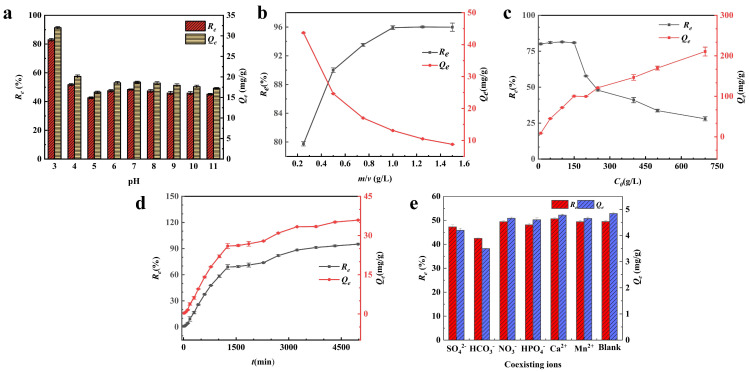
Effects of solution pH (**a**), *m*/*v* ratio (**b**), initial solution concentration (**c**), reaction time (**d**), and coexisting ions (**e**) on the adsorption of Sb(III) by FM@GC.

According to the experimental results, the initial pH of the solution greatly affects the adsorption efficiency and amount of FM@GC, and different initial pH values influence the form of Sb(III) in the water column [22]. Figure 2a shows the variation curves for removal efficiency (*R_e_*) and equilibrium adsorption amount (*Q_e_*) of Sb(III) adsorbed by FM@GC under different pH conditions (3~11). The removal efficiency decreased from 82.91% to 45.09% and the equilibrium adsorption amount decreased from 31.98 mg/g to 17.24 mg/g as the initial pH increased from 3 to 11. As the pH value increases, both the equilibrium adsorption capacity of Sb(III) and the removal efficiency of FM@GC decrease, indicating that the adsorption of Sb(III) by FM@GC is most effective in an acidic environment. When the pH is between 3 and 10, Sb(III) mainly exists as neutral molecules HSbO_2_ and Sb(OH)_3_ [23]. It has been demonstrated that Sb(III) can be adsorbed onto the material’s surface through ligand reactions with reactive functional groups (−OH, −C−O, and C=O) on the adsorbent under acidic conditions [24]. At lower pH levels, the protonation of the active functional groups on the adsorbent surface is enhanced, leading to a stronger adsorption capacity [25]. As the pH increases (i.e., under weakly acidic and alkaline conditions), protonation of the functional groups is less favorable, weakening their adsorption capacity, which leads to a decreased adsorption effect of FM@GC on Sb(III). Consequently, the removal efficiency decreases with increasing pH. The removal efficiency is relatively enhanced at pH 6 to 9 compared to pH 5, possibly because Sb is an amphoteric metal prone to precipitation under alkaline conditions, forming more insoluble Sb(OH)_3_, thus slightly increasing the removal rate [1]. In conclusion, pH = 3 is selected as the optimum pH for subsequent experiments.

#### 3.2.2. Influence of Mass-to-Volume Ratio

To investigate the effect of mass-to-volume ratio (*m*/*v*) on the adsorption of Sb(III) by FM@GC, static adsorption experiments were carried out by setting the ratios of the mass of FM@GC to the volume of Sb(III) solution as 0.25, 0.5, 0.75, 1.0, 1.25, and 1.5 g/L. The results are shown in Figure 2b, which indicate that as the *m*/*v* values increase, the removal efficiency (*R_e_*) increases from 79.74% to 95.98%, while the equilibrium adsorption (*Q_e_*) decreases from 43.70 mg/g to 8.77 mg/g. According to Equations (1) and (2) in Section 2.3, an increase in the *m*/*v* values refers to an increase in the amount of FM@GC dosed, while the initial concentration of Sb(III) remains unchanged. This implies that the active adsorption sites and functional groups in the solution increase with the *m*/*v* values, while the amount of Sb(III) in the solution remains constant. As the *m*/*v* values increases, more active adsorption sites and functional groups increase the contact and reaction probability with Sb(III) in the solution [26]. This results in increased adsorption and removal efficiency and decreased equilibrium adsorption amount of Sb(III) by FM@GC, as also observed in the study by Zhuang et al. [27]. At the *m*/*v* value of 1.0 g/L, the removal efficiency of Sb(III) adsorption by FM@GC is 95.91%, and at higher ratios, the removal efficiency remains nearly the same, but the equilibrium adsorption amount is 13.09 mg/g. Therefore, the optimal *m*/*v* value for Sb(III) adsorption by FM@GC is 1.0 g/L.

#### 3.2.3. Influence of Initial Solution Concentration

The experimental results of Sb(III) adsorption by FM@GC are plotted under experimental conditions with different initial Sb(III) concentrations (*C*_0_ = 5 to 700 mg/L) Figure 2c shows that the curves of removal efficiency (*R_e_*) and equilibrium adsorption capacity (*Q_e_*) exhibit a clear trend with increasing initial concentration. When the initial concentration is *C*_0_ = 5.00 to 150.00 mg/L, the removal efficiency gradually decreases from 80.06% to 28.01%, while the equilibrium adsorption amount increases rapidly from 8.88 mg/g to 100.29 mg/g. The high removal efficiency at this stage can be attributed to the high level of active adsorption sites on the surface of FM@GC relative to the amount of Sb(III) in the solution, allowing most of the Sb(III) to be adsorbed efficiently. However, when the initial concentration increases to the range of *C*_0_ = 200 to 700 mg/L, the removal efficiency decreases from 56.26% to 28.01%, while the equilibrium adsorption increases from 99.59 mg/g to 210.53 mg/g. Despite the continuous increase in equilibrium adsorption, the removal efficiency gradually decreases. This may be because the dosage of FM@GC is fixed, implying that the number of its active adsorption sites and functional groups is also fixed [26]. As the amount of Sb(III) in the solution increases with the initial concentration, these adsorption sites rapidly become saturated, leaving a large amount of unabsorbed Sb(III) in the solution. This situation leads to a high adsorption equilibrium concentration (*C_e_*) but a significant reduction in removal efficiency.

#### 3.2.4. Influence of Adsorption Time

The results of the effect of adsorption time on Sb(III) removal are shown in Figure 2d. It is evident that the removal efficiency (*R_e_*) and adsorption amount (*Q_e_*) of Sb(III) gradually increase with time. However, the growth rate of *R_e_* and *Q_e_* slows after 3240 min, indicating that the equilibrium time of Sb(III) adsorption by FM@GC is around 3240 min. During the adsorption process, *R_e_* and *Q_e_* of Sb(III) by FM@GC increases gradually within the first 10 to 1260 min. This relatively fast adsorption may be due to the numerous unsaturated adsorption sites on the composite surface and the higher concentration of Sb(III) in the solution at the early stage. The removal efficiency (*R_e_*) of Sb(III) adsorbed by FM@GC increases from 68.82% to 83.11% between 1560 and 3240 min, suggesting that the equilibrium time for Sb(III) adsorption by FM@GC is approximately 3240 min. However, the growth rate of both *R_e_* and *Q_e_* slows compared to the earlier stage. This slowdown is likely because many adsorption sites of FM@GC become saturated during the initial stage, and the concentration of Sb(III) in the solution decreases [28]. This reduction leads to a lower probability of contact and reaction between the adsorption sites on FM@GC and Sb(III), resulting in a reduced growth rate. After 3240 min, the Re for Sb(III) by FM@GC increases from 83.11% to 88.56%, but the growth rate further declines, likely because the active adsorption sites on FM@GC are mostly saturated and the concentration of Sb(III) is low, making it difficult for FM@GC to capture Sb(III) in the solution. In summary, the adsorption equilibrium time of Sb(III) by FM@GC is about 3240 min, with an equilibrium adsorption rate exceeding 80%.

#### 3.2.5. Influence of Coexisting Ions

To investigate the influence of common anions and cations in water on the adsorption of Sb(III) by FM@GC, NO_3_^−^, SO_4_^2−^, HPO_4_^2−^, HCO_3_^−^, Ca^2+^, and Mn^2+^ ions with a concentration of 10.0 mM were added to an Sb(III) solution for adsorption experiments. The results are shown in Figure 2e. Comparing the adsorption results with the blank group without coexisting ions (*R_e_* = 49.46%), HCO_3_^−^ shows the strongest inhibitory effect, reducing the adsorption rate by 7.00%. The effect of HCO_3_^−^ on Sb(III) adsorption may be due to the formation of inner-sphere complexes with iron oxides. SO_4_^2−^ and HPO_4_^2−^ decrease *R_e_* by 1.18% and 2.21%, respectively, indicating slight inhibition, similar to the findings of Deng et al. [29]. Ca^2+^ shows a slight promoting effect, increasing *R_e_* by about 1.20%. The addition of Mn^2+^ and NO_3_^−^ results in very little change in the *R_e_* of Sb(III) adsorbed by FM@GC. It is theorized that the presence of high concentrations of Ca^2+^, Mn^2+^, and Mg^2+^ enhances the adsorption of Sb(III), probably because the high concentration of cations increases the positive charges on the adsorbent surface [1]. This enhancement strengthens the electrostatic interaction between the adsorbent and the Sb(OH)_6_^−^ anion, promoting Sb(III) adsorption. However, compared to previous studies, the above ions have little effect on the adsorption of Sb(III) by FM@GC [12,30].

### 3.3. Adsorption Characteristics

#### 3.3.1. Adsorption Kinetic

**Figure 3:**
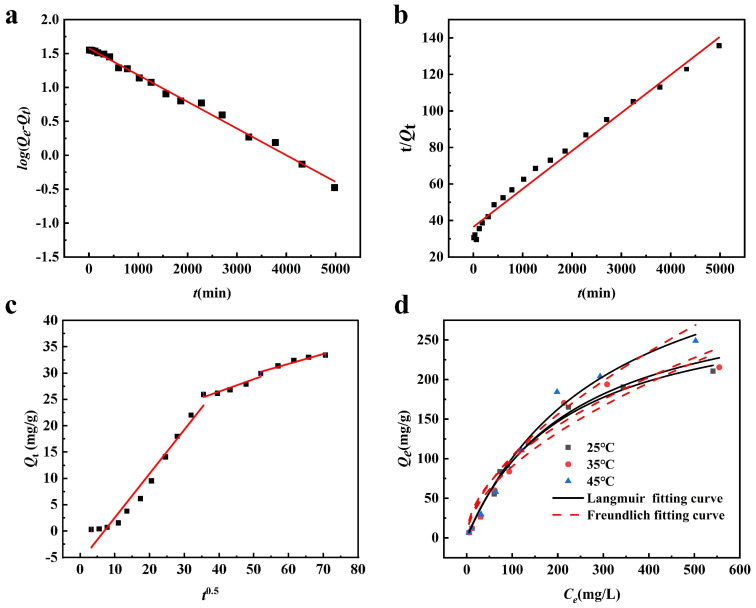
Fitting of adsorption kinetics models for FM@GC adsorption of Sb(III): (**a**) pseudo-first-order kinetics model; (**b**) pseudo-second-order kinetics model; (**c**) Weber–Morris intraparticle diffusion model; and (**d**) isothermal adsorption model fitting.

To investigate the adsorption rate and behavior of Sb(III) on FM@GC during the adsorption process, pseudo-first-order and pseudo-second-order kinetic models were fitted using experimental data. The fitting results of each adsorption kinetic model are shown in Figure 3a,b, and the relevant parameters are listed in Table 1. According to the fitting results, the pseudo-second-order coefficient of determination (*R*^2^) is 0.99, and the fitted *Q_e_* value (36.48 mg/g) is closer to the experimental result (31.37 mg/g). Therefore, the adsorption process of Sb(III) on FM@GC aligns more closely with the pseudo-second-order kinetic model, indicating that the adsorption is predominantly chemical. The experimental data are fitted by the Weber–Morris intraparticle diffusion model, yielding Figure 3c, which shows a multilinear relationship of *Q_t_* versus *t*^0.5^. Sb(III) diffuses rapidly on the surface of FM@GC before slowly diffusing into its pores. The fitted line does not pass through the origin, indicating that intraparticle diffusion is not the sole rate-controlling mechanism and that the adsorption process of Sb(III) on FM@GC involves multiple mechanisms [31].

#### 3.3.2. Isothermal Adsorption

Figure 3d presents the experimental results of FM@GC isothermal adsorption of Sb(III) at 25 °C, 35 °C, and 45 °C, along with the fitting curves for the Langmuir and Freundlich models. The relevant parameters are listed in the table. It is observed that under constant temperature conditions, the equilibrium adsorption capacity *(Q_e_*) increases with the equilibrium concentration (*C_e_*). As the temperature rises, *Q_e_* also increases, indicating that the adsorption capacity of FM@GC for Sb(III) is enhanced with higher temperatures. The fitting results indicate that the Langmuir model shows a poor fit at various temperatures, with a coefficient of determination (*R*^2^) of 0.83, and a maximum adsorption capacity (*Q_m_*) for Sb(III) at 25 °C of 178.89 mg/g. In contrast, the Freundlich model demonstrates a better fit, with the *R*^2^ = 0.93, indicating that the adsorption process of FM@GC for Sb(III) is primarily multilayer, with uneven distribution of adsorption sites on its surface. The parameters *K_F_* and 1/*n* are related to the adsorbent, adsorption mechanism, and reaction temperature. Studies show that when *1*/*n* < 0.5, the adsorbent is stably adsorbed by the material [27]. According to the calculated results of Equation (4), Table 2 shows that at 25 °C, the values of 1/*n* and *K_F_* are 0.36 and 23.68, respectively, indicating that FM@GC can stably adsorb Sb(III) in solution.

### 3.4. Adsorption Mechanism

**Figure 4:**
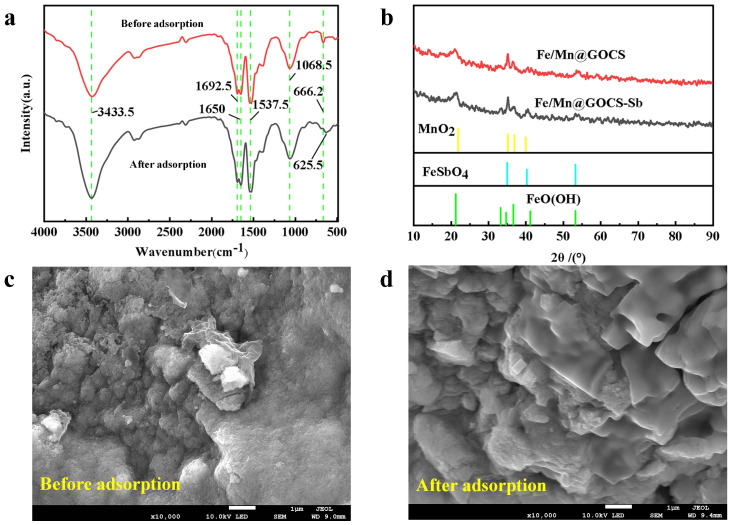
FTIR (**a**), XRD (**b**) and SEM patterns of FM@GC before (**c**) and after (**d**) adsorption of Sb(III).

Figure 4a shows the FTIR spectra of FM@GC before and after the adsorption of Sb(III). The characteristic peak at 3433.5 cm^−1^ is the O–H stretching vibration peak, indicating the presence of hydroxyl groups in the material. The 3433.5 cm^−1^ peak is enhanced after adsorption, probably because Sb(III) forms intersperse complexes with –OH [2]. This suggests that the Sb(III) adsorption process is related to O–H. The characteristic peaks near 1692.5–1650 cm^−1^ may be due to the C=O stretching vibration in –NHCO– and the N–H absorption of –NH_2_, suggesting the presence of amide bonds in the material [32]. The peak at 1537.5 cm^−1^, attributed to C=C vibration, and the peak at 1068.5 cm^−1^, corresponding to C–O stretching vibration, also exhibit changes post-adsorption [33]. This suggests the participation of these functional groups in the adsorption mechanism, which is consistent with the study by Simić et al. [34], where shifts in C–O and C=O bands are observed during the adsorption of metal ions, indicative of chemical interactions such as ligand-exchange and chemisorption. Moreover, the shift of the characteristic peak from 666.2 cm^−1^ before adsorption to 625.5 cm^−1^ after adsorption indicates the involvement of α–FeO(OH) and Mn–O in the adsorption process of Sb(III). Such shifts in FTIR spectra, particularly involving metal-oxygen bonds, have been similarly reported in the literature as evidence of direct interactions between the adsorbate and the adsorbent surface, further supporting the proposed adsorption mechanism [35,36].

After the adsorption of Sb(III), key changes are observed in the FM@GC composite material (Figure 4b). Initially, a new characteristic peak at 2*θ* = 40.53°, similar to the XRD spectrum of FeSbO_4_, indicates that Sb(III) is successfully adsorbed onto the surface of the composite material, potentially forming an FeSbO_4_-type compound, as observed in the study by Zhang et al. [37]. The formation of this mineral phase is crucial evidence for the removal and stabilization of Sb(III) from the solution into the solid phase. Furthermore, the increased intensity of the FeO(OH) characteristic peak at 2*θ* = 33.48° supports the active role of the FeO(OH) groups during the adsorption process of Sb(III) [38]. This enhanced signal suggests that the FeO(OH) functional group is associated with the removal of Sb(III). The presence of characteristic peaks of MnO_2_ at 2*θ* = 37° and 54° indicates that Mn(II) is partially oxidized during adsorption [39]. This suggests that partial oxidation of Mn(II) occurs during the adsorption process. MnSO_4_ might be influenced by the oxidative environment during the adsorption of Sb(III), where Mn(II) is oxidized to Mn(IV), forming MnO_2_. This oxidation could be due to the oxidative properties of graphene oxide (GO) or the presence of oxidants in the experimental conditions, such as oxygen in the air or other oxidative media [40]. The formation of MnO_2_ not only alters the chemical structure of the material but also might increase the adsorptive active sites, thereby enhancing the efficiency of Sb(III) adsorption. The formation of MnO_2_ could facilitate the oxidation of Sb(III) to a more stable Sb(V), further removed through specific adsorptive sites of MnO_2_ or FeO(OH) oxidation products [37]. The appearance of these MnO_2_ characteristic peaks, along with the enhancement of FeSbO_4_ and FeO(OH) peaks, indicates that complex chemical reactions are simultaneously occurring on the material surface, leading to the effective removal and stabilization of Sb(III).

After Sb(III) adsorption, as shown in the SEM image in Figure 4c, the spherical surface of the composite material became smooth with no visible pores. Sheet-like or scaly materials and flocculent substances were attached to the surface of the spherical particles. The surface changes of FM@GC after Sb(III) adsorption are likely related to the formation of antimony-containing complexes.

As shown in the EDS energy spectrum before and after adsorption (Table 3), the elements with high content in the FM@GC composite before Sb(III) adsorption are C (22.82%), O (39.99%), and Fe (34.99%), while the elements with low content are N (1.72%) and Mn (0.48%). After Sb(III) adsorption by the FM@GC composite, the content of C and N decreases by 4.46% and 0.23%, respectively, while the content of Mn increases slightly. The content of Fe increases significantly to 55.60%, the content of O decreases significantly to 19.35%, and Sb is 5.5%. These results suggest that the Mn-O group within FM@GC plays a critical role in the oxidation of Sb(III) during the adsorption process, while the Fe-O group serves as the main adsorption site for Sb. This observation aligns with findings from previous studies, where it was observed that Mn-O groups in FM@GC act as oxidation sites for Sb(III), and Fe-OOH groups are identified as primary adsorption sites [2]. The oxidation of Sb(III) to Sb(V) by Mn-O groups enhances the adsorption capacity of the composite, as Sb(V) species, particularly those with negative charges, are more readily adsorbed onto metal oxides than Sb(OH)_3_, the predominant species of Sb(III) in weakly acidic to neutral pH conditions [2]. Antimony-containing compounds such as FeSbO_4_ are generated, which is consistent with the XRD analysis results. In summary, the adsorption process of Sb(III) by FM@GC mainly involves converting Sb(III) in water to Sb(V) through oxidation by the Mn-O group, and then forming a complex with O-H and Sb in the FeO(OH) group through ligand exchange [3]. The FTIR analysis corroborates this mechanism, suggesting that Sb(III) is first oxidized and then adsorbed onto the composite material through chemical interactions.

## 4. Conclusions

In this study, four different forms of MnO_x_—MnSO_4_, MnCl_2_, KMnO_4_, and MnO_2_—were used to chemically modify Fe-graphene–chitosan (GOCS) composites. Four composites—MnSO_4_/Fe@GC, MnCl_2_/Fe@GC, KMnO_4_/Fe@GC, and MnO_2_/Fe@GC—were prepared. The adsorption properties of these materials were systematically studied for Sb(III). Among all the tested composites, MnSO_4_/Fe@GC showed the best adsorption performance for Sb(III). This suggests that MnSO_4_ combines with Fe@GOCS more effectively than other MnO_x_, possibly because these two compounds form more stable chemical bonds on the GOCS matrix. The adsorption capacity of Sb(III) by FM@GC increases with the initial Sb(III) concentration, but the removal rate decreases. In addition, as the *m*/*v* value increases, the adsorption capacity gradually decreases, while the removal efficiency increases, indicating that the optimal *m*/*v* value is 1.0 g/L. The adsorption performance of the material is better under acidic conditions and decreases significantly as the pH increases. It takes 3420 min for FM@GC to reach adsorption equilibrium, and the coexisting ions have little effect on the adsorption of Sb(III). The adsorption of Sb(III) by FM@GC aligns more closely with the pseudo-second-order kinetic model, with a coefficient of determination *R*^2^ = 0.99. The experimental data fit the model very closely, indicating that chemisorption plays a leading role in the removal process of Sb(III). The Langmuir model fits poorly, while the Freundlich model fits better, emphasizing the multilayer adsorption process on heterogeneous surfaces. XRD and SEM-EDS indicate that FM@GC is a porous microspherical structure, where the Mn-O group promotes the oxidation of Sb(III), and the FeO(OH) group serves as the main adsorption site for Sb.

## Figures and Tables

**Table 1:** Adsorption kinetics model parameters.

Temperature (°C)	Pseudo First Order			Pseudo Second Order		
	*Q_e_* (mg/g)	*k*_1_	*R* ^2^	*Q_e_* (mg/g)	*k*_2_	*R* ^2^
25	1.58	−0.00039	0.9817	36.48	0.020	0.9923

**Table 2:** Langmuir and Freundlich fitting data table.

Temperature (°C)	Langmuir Model			Freundlich Model		
	*Q_e_* (mg/g)	*K_L_*	*R* ^2^	*K* *_F_*	*1*/*n*	*R* ^2^
25	178.89	0.0335	0.8430	24.55	0.34	0.9306
35	214.09	0.0223	0.8205	21.91	0.38	0.9139
45	226.35	0.0238	0.8293	24.58	0.37	0.9328

**Table 3:** The EDS energy spectrum before and after adsorption.

Elements	C	Fe	O	Mn	Sb
Before adsorption	22.82	34.99	39.99	0.48	-
After adsorption	18.36	55.60	19.35	1.19	5.5

## Data Availability

Data is contained within the article.
